# A Patency Capsule Remained Intact in the Colon over 210 Hours

**DOI:** 10.1155/2017/1201404

**Published:** 2017-02-22

**Authors:** Yu Hihara, Satoru Joshita, Toshiharu Takahashi, Shinji Okaniwa, Yoshiki Mizukami, Yoshiyuki Nakamura

**Affiliations:** ^1^Department of Gastroenterology, Iida Municipal Hospital, Iida, Japan; ^2^Department of Medicine, Division of Gastroenterology and Hepatology, Shinshu University School of Medicine, Matsumoto, Japan; ^3^Department of Surgery, Iida Municipal Hospital, Iida, Japan

## Abstract

We present an unusual case of a 35-year-old male patient whom a patency capsule stayed in his gut without breaking. He has a history of Peutz-Jeghers syndrome and multiple abdominal surgeries. Prestudy was performed for abdominal searching, but a patency capsule remained in the colon over 9 days. He displayed neither abdominal nor obstructive symptoms in that period. We collected the patency capsule using colonoscopy after dilating a postoperative stricture at an anastomotic site of the rectum. Clinicians should bear in mind that patency capsules may become retained as distally as the colon in patients with a surgical history of the large intestine.

## 1. Introduction

Prestudy using a patency capsule is advised prior to regular capsule endoscopy [1], especially for patients who are suspected of having a stenotic lesion in the gut, because the capsule is dissolvable and naturally broken down within 100–200 hours. However, instances of retainment in the small intestine have been reported.

Herein, we describe a patient who showed the patency capsule's retainment in the colon.

## 2. Case Report

**Figure 1:**
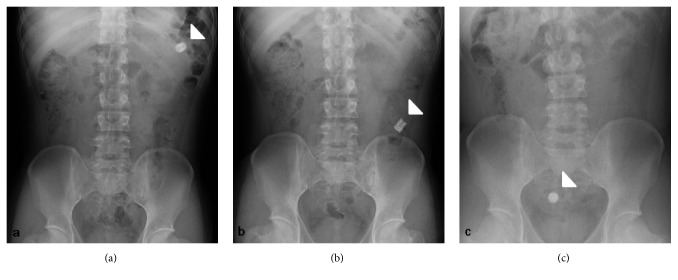
Abdominal radiographs taken at (a) 31, (b) 56, and (c) 218 hours after ingestion showing the patency capsule to be moving anally, but finally becoming retained in the right iliac fossa (arrowheads).

**Figure 2:**
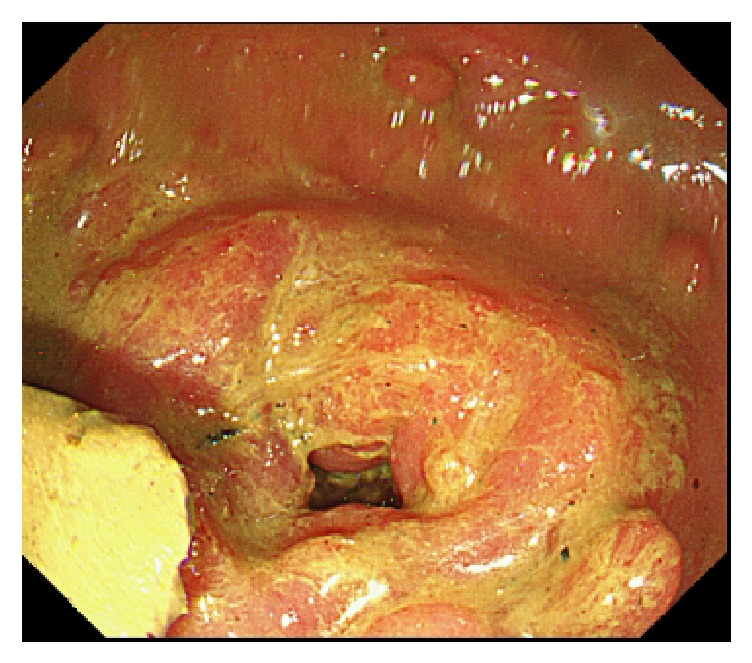
Colonoscopy displaying the anastomotic stenosis between the rectum and the sigmoid colon.

**Figure 3:**
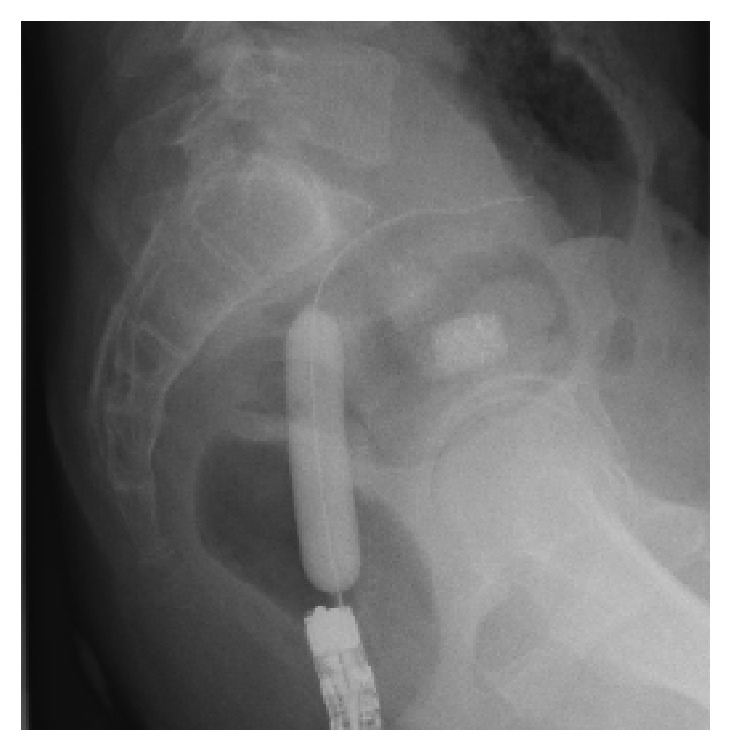
Abdominal radiograph depicting the endoscopic balloon dilatation for the stenotic lesion between the rectum and the sigmoid colon.

**Figure 4:**
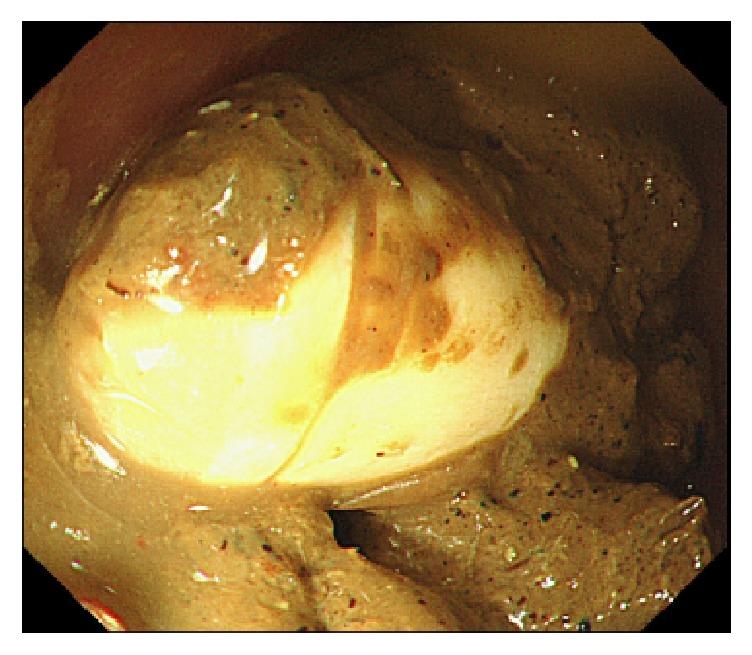
The patency capsule was found to be surrounded by colonic feces.

**Figure 5:**
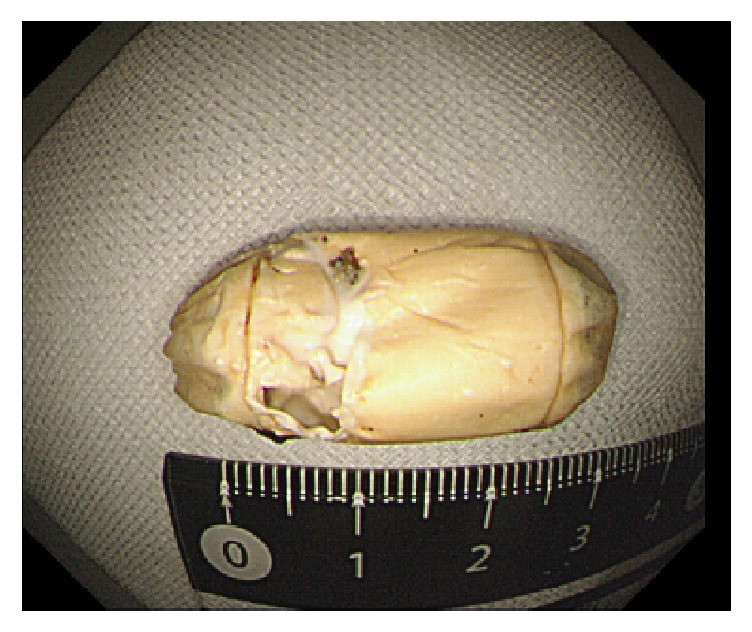
The patency capsule was collected from the colon by colonoscopy.

A 35-year-old man was diagnosed as having Peutz-Jeghers syndrome at the age of 7 years. Since he had undergone 3 polypectomy procedures by laparotomy of the small intestine, the latter of which with low anterior resection for rectum cancer, we began prestudy using a patency capsule in preparation for a capsule endoscopy to screen for polyps in the small intestine. He did not receive a colonoscopy just prior to prestudy with the patency capsule. The capsule was visible in the left upper abdomen at 31 hours after ingestion by radiography (Figure 1(a)) and had moved to the lower abdomen at 56 hours (Figure 1(b)). We observed the patient carefully over the following days as the capsule moved slowly, but was not discharged. Surprisingly, the patency capsule was lodged in the right iliac fossa after 218 hours (Figure 1(c)). The patient displayed neither abdominal nor obstructive symptoms. Colonoscopy carried out at 223 hours (at 9 days) after ingestion of the patency capsule revealed severe stenosis of a postoperative stricture at an anastomotic site of the rectum (Figure 2). We conducted endoscopic balloon dilatation of the stenotic lesion using a 12–15 mm balloon via colonoscopy (Figure 3) and could pass the colonoscope through the stenosis. The patency capsule was found to be covered by feces at the blind-ending colon (Figure 4) but was easily and successfully collected (Figure 5) to reveal that the body and plugs were virtually intact.

## 3. Discussion

Prestudy using a patency capsule is advised prior to regular capsule endoscopy, especially for patients who are suspected of having a stenotic lesion in the gut [2, 3], because the capsule is dissolvable and naturally broken down within 100–200 hours. Capsules are normally discharged without complications, but instances of retainment in the small intestine have been reported [4, 5]. To our knowledge, this is the first case of a patency capsule retained in a blinded area in the colon that allowed feces to pass through a stenosis and avoid obstructive symptoms despite being retained for an extended time period (i.e., over 9 days). Although feces are also known to contain bacteria that produce lactase, we cannot exclude the possibility that the patency capsule was protected by the feces from contact with intestinal fluids, which might explain how it remained virtually intact. Clinicians should bear in mind that patency capsules may become retained as distally as the colon in patients with a surgical history of the large intestine.
